# Evaluation of radiophotoluminescent glass dosimeter response for therapeutic spot scanning proton beam: suggestion of linear energy transfer‐based correction

**DOI:** 10.1002/acm2.13378

**Published:** 2021-08-02

**Authors:** Junya Nagata, Keisuke Yasui, Chihiro Omachi, Toshito Toshiyuki, Hidetoshi Shimizu, Takahiro Aoyama, Naoki Hayashi

**Affiliations:** ^1^ Graduate School of Health Sciences Fujita Health University Toyoake Japan; ^2^ Faculty of Radiological Technology School of Health Sciences Fujita Health University Toyoake Japan; ^3^ Nagoya Proton Therapy Center Nagoya City West Medical Center Nagoya Japan; ^4^ Department of Radiation Oncology Aichi Cancer Center Hospital Nagoya Japan

**Keywords:** glass dosimeter, linear energy transfer, postal audit, proton dosimetry, under‐response correction

## Abstract

A radiophotoluminescent glass dosimeter (RGD) is used for a postal audit of a photon beam because of its various excellent characteristics. However, it has not been used for scanning proton beams because its response characteristics have not been verified. In this study, the response of RGD to scanning protons was investigated to develop a dosimetry protocol using the linear energy transfer (LET)‐based correction factor. The responses of RGD to four maximum‐range‐energy‐pattern proton beams were verified by comparing it with ionization chamber (IC) dosimetry. The LET at each measurement depth was calculated via Monte Carlo (MC) simulation. The LET correction factor ($k_{\mathrm{LET}}^{\mathrm{RGD}}$) was the ratio between the uncorrected RGD dose ($D_{\mathrm{raw}}^{\mathrm{RGD}}$) and the IC dose at each measurement depth. $k_{\mathrm{LET}}^{\mathrm{RGD}}$ can be represented as a function of LET using the following equation: $k_{\mathrm{LET}}^{\mathrm{RGD}}\left(\mathrm{LET}\right)=‐0.035\left(\mathrm{LET}\right)+1.090$. $D_{\mathrm{raw}}^{\mathrm{RGD}}$ showed a linear under‐response with increasing LET, and the maximum dose difference between the IC dose and $D_{\mathrm{raw}}^{\mathrm{RGD}}$ was 15.2% at an LET of 6.07 keV/μm. The LET‐based correction dose ($D_{\mathrm{LET}}^{\mathrm{RGD}}$) conformed within 3.6% of the IC dose. The mean dose difference (±SD) of $D_{\mathrm{raw}}^{\mathrm{RGD}}$ and $D_{\mathrm{LET}}^{\mathrm{RGD}}$ was –2.5 ± 6.9% and 0.0 ± 1.6%, respectively. To achieve accurate dose verification for scanning proton beams using RGD, we derived a linear regression equation based on LET. The results show that with appropriate LET correction, RGD can be used for dose verification of scanning proton beams.

## 1 INTRODUCTION

Determining the absorbed dose to water affects radiotherapy and requires accurate dosimetry and quality assurance (QA).^1^, ^2^, ^3^ Therefore, external dosimetry audits are crucial, and, in Japan, a radiophotoluminescent glass dosimeter (RGD) is used for postal audit in a photon beam.^4^ Dose measurement using the RGD has been established for a photon beam even under non‐reference conditions with wedges and intensity‐modulated radiation therapy (IMRT), thus contributing to the realization of safe and highly accurate radiation therapy.^5^, ^6^

An external dosimetry audit was performed at the proton therapy facility in the U.S. by inserting thermoluminescent dosimeters (TLDs) into five anthropomorphic phantoms.^7^ A dose accuracy of less than 5% in a region with small changes in linear energy transfer (LET) and 7% over‐response has been reported for TLD dosimeters, except in the distal region.^8^, ^9^, ^10^ For carbon‐ion beams in Japan, an external dosimetry audit has been devised to visit carbon‐ion beam facilities and evaluate the output using an ionization chamber (IC)^11^; however, owing to the difference in the number of proton and carbon beam facilities in Japan (18 proton facilities and 6 carbon facilities), it is difficult to implement the external audit for proton facilities. On the other hand, RGD used for an external audit in Japan exhibits less fading (within 1.7% after five months) and can be read repeatedly.^12^ In addition, RGD has excellent dose characteristics; good dose linearity and energy dependence are reported to be approximately 1.6% for 6 and 15 MV photon beams, respectively, and the sensitivity volume is small.^12^ Because of these characteristics, as mentioned above, postal audits using RGD have been established for photon beams, and it is necessary to establish a proton dosimetry protocol using RGD.

The RGD response of proton beams has been verified in various reports using passive methods.^13^, ^14^, ^15^, ^16^ Rah et al. defined the LET dependence of RGD as a second polynomial in the range of residual range (R_res_) from 2.1 to 9.0 cm for a proton beam.^16^ Chang et al. investigated the correction using R_res_ of the stopping power ratio (SPR) and LET quenching for the RGD measurement of a 200 MeV proton beam.^14^ In these two studies, the beam quality was defined using R_res_, and a correction formula was derived. However, the beam quality of proton beams is significantly affected by LET, which depends on the energy and spread‐out Bragg peak (SOBP) width. Therefore, LET is not accurately indicated by R_res_. In our previous study, we used LET calculated through Monte Carlo (MC) simulation to correct the RGD response for the 100–225 MeV passive proton beam system.^13^ A 15.7% dose reduction of RGD owing to LET was observed for passive proton beams, and the mean dose difference (±SD) was improved at 0.0 ±2.1% after LET‐based correction.^13^ Previous studies have shown the response of RGD to proton beams with LET‐based corrections. However, the RGD response to scanning proton beams has not yet been investigated.

Most facilities for proton therapy constructed in recent years have adopted a scanning system;^17^, ^18^ therefore, the response characteristics of RGD to the scanning proton beam are necessary to perform accurate dosimetry and establish a postal audit using the RGD. This study aims to evaluate the RGD response and devise an appropriate RGD dosimetry protocol using LET‐based correction for scanning proton beams. MC was used to calculate the LET for each depth of the scanning proton beam. For this purpose, the RGD and IC doses were compared at each measurement depth and the correlation with the LET calculated by MC was verified.

## 2 MATERIALS AND METHODS

### 2.1 Dosimetry system for RGD

**FIGURE 1:**
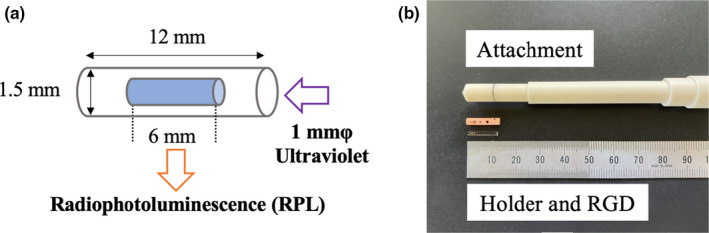
(a) Details of the geometric information and (b) measurement attachment of the RGD

In this study, we used RGDs (GD‐302 M, Asahi Techno Glass, Japan) and an automatic reader (FGD‐1000, Asahi Techno Glass, Japan). Figure 1 demonstrates the geometric information and measurement attachment of the RGD reading. RGDs have excellent characteristics, such as uniformity, reproducibility, and dose linearity. The energy dependence is within 2% for ^60^Co γ‐rays as well as 6 and 10 MV photon beams, and the direction dependence is within 1.5% from 0° to 180° for the long‐axis direction of the RGD.^19^ RGD is a silver‐activated phosphate glass element, and its weight composition is as follows: Na (11.0%), P (31.55%), O (51.16%), Al (6.12%), and Ag (0.17%).^20^ Its effective atomic number and density are 12.04 and 2.61 g/cm^3^, respectively.^21^ Additionally, it forms radiophotoluminescence (RPL) centers after irradiation, which are excited by ultraviolet irradiation and release RPL. The amount of RPL was proportional to the absorbed dose irradiated.

The RGD used in this study had a diameter and length of 1.5 and 12 mm, respectively, with a serial number. The readout volume had a diameter and length of 1 and 6 mm, respectively, and the readout center was in the opposite direction of the serial number marked on the RGD.^22^ To promote the formation of RPL centers, the RGDs were annealed at 400℃ for 1 hour before they were measurement and preheated (70℃ for 30 minute) after proton beam irradiation.

### 2.2 **Dosimetry and** $k_{\text{LET}}^{\text{RGD}}$

**FIGURE 2:**
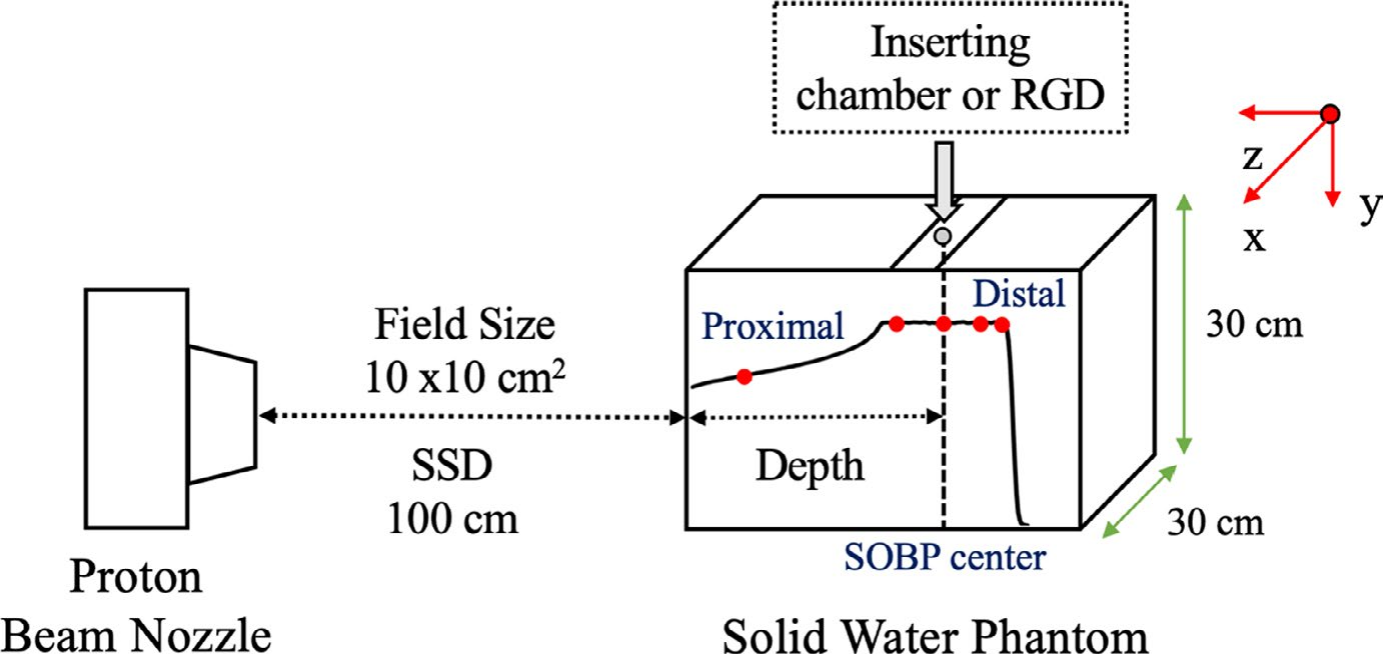
Geometric information of the measurement and the measurement depth at the SOBP. The red dots represent the measurement depth and defined proximal, SOBP center, and distal region

In this study, we used a proton beam delivery system PROBEAT‐III (Hitachi, Ltd., Japan) at the Nagoya Proton Therapy Center (NPTC). For a spot scanning proton beam system, 95 energies from the synchrotron and a maximum range of 4–30.6 g/cm^2^ were available.^23^ An energy absorber and collimator were used to irradiate the shallow regions of less than 4 g/cm^2^, and the spot sizes ranged from 4.7 to 13.8 mm.^24^, ^25^ Figure 2 shows the geometric information of the measurement and measurement depth. We used proton beams with four maximum range energy patterns (80, 120, 200, and 300 mm), and the absolute dose was approximately 2 Gy at the center of SOBP. The modulated SOBP widths were 40 (range; 80–120 mm) and 90 mm (range; 200–300 mm). The measurement depths are 20 mm and proximal, central, and distal to the SOBP. The field size at the SOBP center was 10 × 10 cm^2^. The dose measurements were performed using a pinpoint 3D IC (model TM31016, PTW, Germany) and RGDs. The radius and length of the pinpoint 3D IC were 1.45 and 2.9 mm, respectively. The measurements were repeated thrice, and the average value was selected. In this study, we used the SOBP center of the 104.5–130.5 MeV/SOBP 40 mm (range: 120 mm) proton beam as the reference condition for convenience. The absolute dose to water of RGD was calibrated using the conversion factor ($N_{D,w}^{\mathrm{RGD}}$) with an IC dose under the reference conditions. The depth‐dose distributions acquired during commissioning were used to evaluate the RGD response. The RGD response was the ratio between the uncorrected RGD dose ($D_{\mathrm{raw}}^{\mathrm{RGD}}$) and the IC dose at each measurement depth, in which four maximum range energy pattern proton beams were used. $D_{\mathrm{raw}}^{\mathrm{RGD}}$ is expressed using the following equation:

(1) $$D_{\mathrm{raw}}^{\mathrm{RGD}}=\;M_{\mathrm{raw}}^{\mathrm{RGD}}\times N_{D,w}^{\mathrm{RGD}},$$

where $M_{\mathrm{raw}}^{\mathrm{RGD}}$ is the RPL and $N_{D,w}^{\mathrm{RGD}}$ is the conversion factor. An approximate curve of the RGD response was produced as a function of LET and used as the LET correction factor ($k_{\mathrm{LET}}^{\mathrm{RGD}}$). The RGD dose of the LET‐based correction ($D_{\mathrm{LET}}^{\mathrm{RGD}}$) was obtained by multiplying the RPL by the reciprocal of $k_{\mathrm{LET}}^{\mathrm{RGD}}$. $D_{\mathrm{LET}}^{\mathrm{RGD}}$ is expressed as follows:

(2) $${D_{\mathrm{LET}}^{\mathrm{RGD}}=M}_{\mathrm{raw}}^{\mathrm{RGD}}\times \frac{1}{k_{\mathrm{LET}}^{\mathrm{RGD}}},$$

where $M_{\mathrm{raw}}^{\mathrm{RGD}}$ is the RPL and $k_{\mathrm{LET}}^{\mathrm{RGD}}$ is the LET correction factor which varies with the LET of the measurement depth. The depth‐dose distributions calculated using treatment planning system (TPS) were used to evaluate the RGD response.

### 2.3 LET calculated via MC simulation

Here, we used a particle therapy simulation framework (PTSIM) based on MC code GEANT4 version 10.05.p01.^26^, ^27^, ^28^ PTSIM is an MC package developed for simulation of particle beam transport. The geometry of a delivery system nozzle of a spot‐scanning proton at NPTC was simulated using PTSIM, and the average LET in water was calculated. The average LET is categorized into two types: track‐averaged LET (LETt) and dose‐averaged LET (LETd). Because LETd is directly connected with dose, it is a suitable physical quantity for evaluating the response of a detector to a proton beam.^29^ Therefore, we used LETd for an analysis in this study. LET is expressed as follows:

(3) $$\mathrm{LET}\left(z\right)=\frac{\int \Phi_{E}\left(z\right){\mathrm{LET}}^{2}\left(E\right)dE}{\int \Phi_{E}\left(z\right)\mathrm{LET}\left(E\right)dE},$$

where Φ*_E_* (*z*) is the fluence of the proton beam with energy *E* at depth *z*, and $\mathrm{LET}\left(E\right)$ is the LET of the proton beam with energy *E*.

Based on the MC calculations, we used the same parameters as in a previous report.^13^ The physics lists G4mStandardPhysics_option4 and G4HadronPhysicsQGSP_BIC were used for simulating proton and nuclear interactions, respectively. The detection volume of the LET was 12 × 3 × 1.5 mm^3^, which corresponds to the actual RGD dimensions (diameter 1.5 mm, length 12 mm), although the lateral dimensions were expanded sufficiently to account for the uncertainty of MC calculation. The statistical uncertainty of the MC calculation to obtain the LET was within 5%.

## 3 RESULTS

### 3.1 Calclation of [described equation]

#### 3.1.1 $k_{LET}^{RGD}$

**FIGURE 3:**
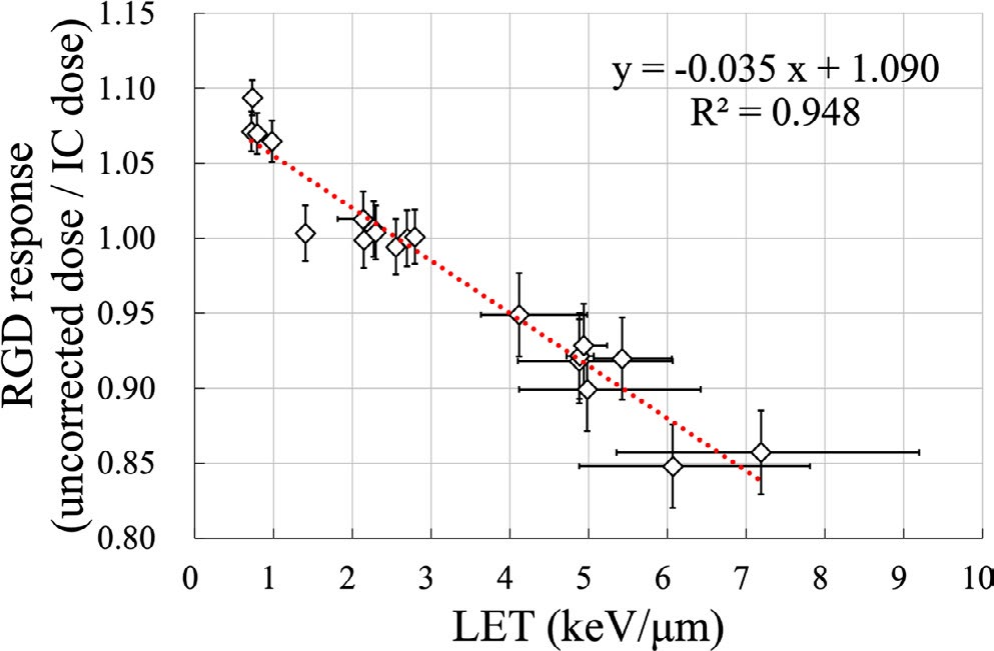
RGD response as a function of LET for a spot scanning proton beam. IC dose is the dose of chamber measurement. The vertical error bars indicate the uncertainty of the absolute dose. The horizontal error bars indicate the change in LET as measurement depth is shifted by ±2 mm. The dotted line represents $k_{\mathrm{LET}}^{\mathrm{RGD}}$ for the LET fitted using a linear function

Figure 3 demonstrates the RGD response as a function of LET calculated via MC simulation. The dotted line represents the RGD response fitted by a LET linear function. The LET for the reference condition was 2.7 keV/μm. RGD response as a function of LET is expressed using the following equation:

(4) $$k_{\mathrm{LET}}^{\mathrm{RGD}}\left(\mathrm{LET}\right)=‐0.035\left(\mathrm{LET}\right)+1.090,$$

where $k_{\mathrm{LET}}^{\mathrm{RGD}}$ is the LET correction factor which varies with the LET of the measurement depth. The corrected dose for the RGD quenching effect was obtained by multiplying the reciprocal of $k_{\mathrm{LET}}^{\mathrm{RGD}}$ with RPL. The vertical error bars in Figure 3 indicate the uncertainty of the measurement dose (±3% in the distal region and ±2% in other regions based on our previous study). The horizontal error bars indicate the positional uncertainty in the measurement depth in LET. The LET was increased in the distal region as in a previous study.^13^ For a measurement depth uncertainty of ±2 mm, the resulting LET uncertainty in the plateau region is approximately 0.01 keV/μm and the maximum LET uncertainty in the distal region is 2.01 keV/μm. The effect of the positional uncertainty was large in the distal region.

### 3.2 Correlation between R_res_ and LET

**FIGURE 4:**
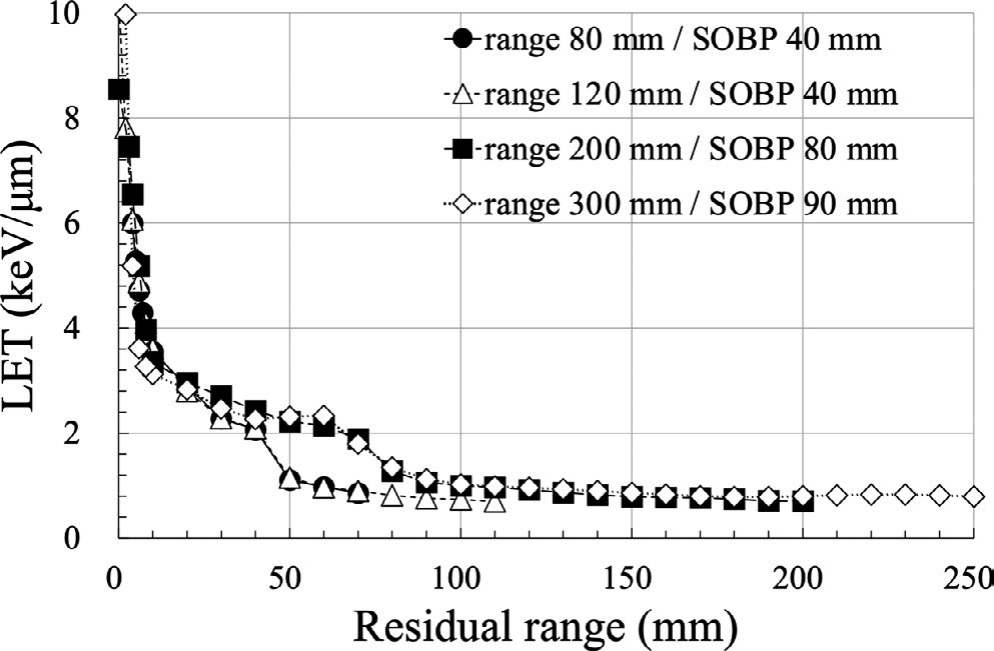
Relationship between Rres and LET obtained via MC simulation for the scanning proton beams with four maximum range energy patterns

Figure 4 shows the relationship between R_res_ and LET obtained via MC simulation for proton beams with four maximum range energy patterns. The LET values for the 100–225 MeV passive proton beam have been reported to range from 10% to 30% for a similar R_res_.^13^ In the scanning SOBP, the LET value suddenly increased for R_res_ <20 mm and varied from 10% to 50% in the range of R_res_ 40–80 mm. Therefore, the LET values were different for a similar R_res_, and the LET varied with irradiation conditions, such as energy and SOBP width. The amount of change in LET for a spot scanning system increased compared with that of a passive system. Figure 4 shows that the LET value increased in the entrance region of SOBP (at 40–50 mm‐R_res_ for ranges 80 and 120 mm, at 70–80 mm‐R_res_ for ranges of 200 and 300 mm), which is different from that of a passive system.

### 3.3 Details of RGD response for spot‐scanning proton beam

**FIGURE 5:**
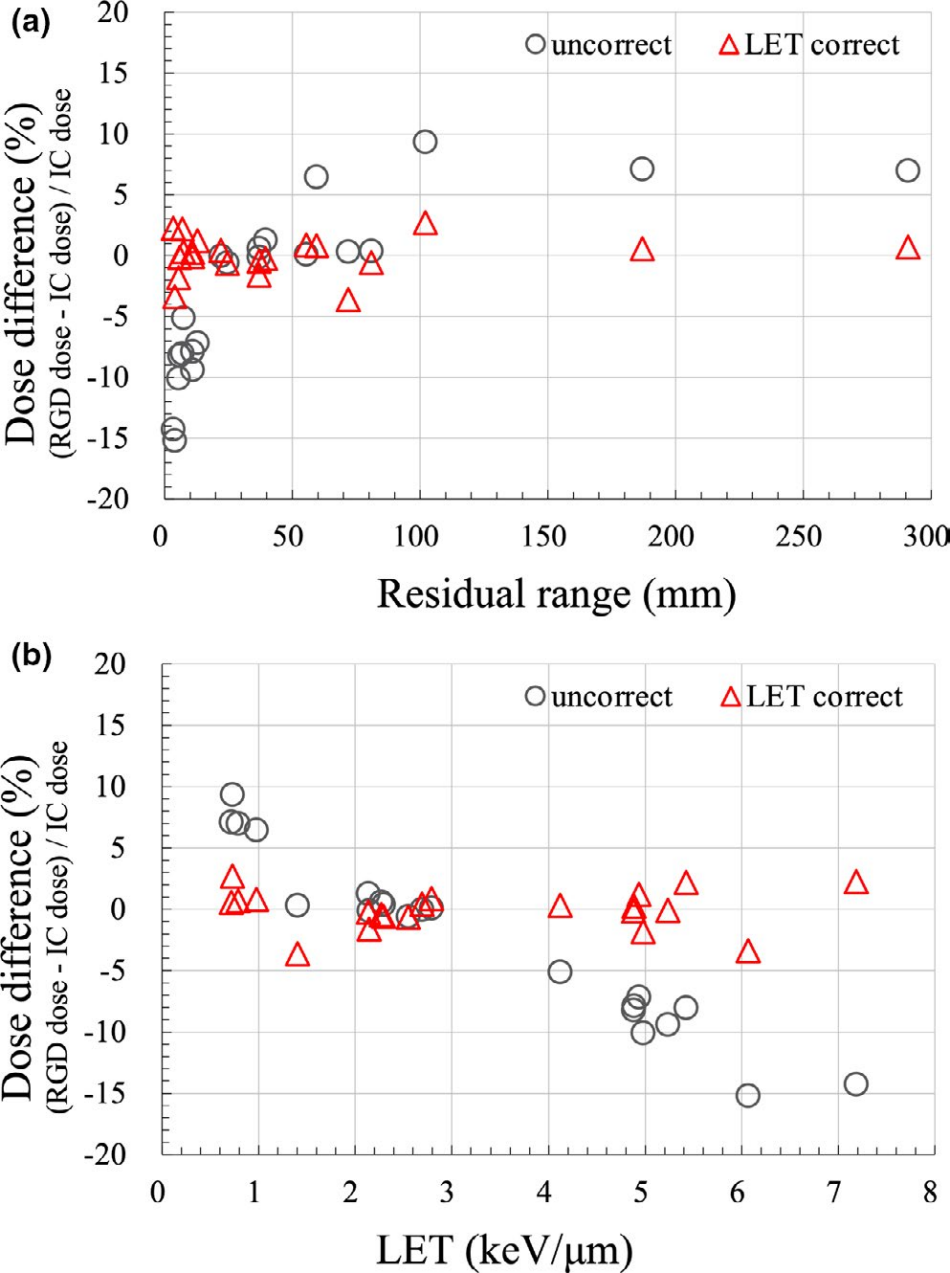
Dose difference between the IC and RGD as a function of (a) Rres and (bs) LET. The gray circles and red triangles represent uncorrected and corrected doses, respectively. IC dose is the dose of chamber measurement

**FIGURE 6:**
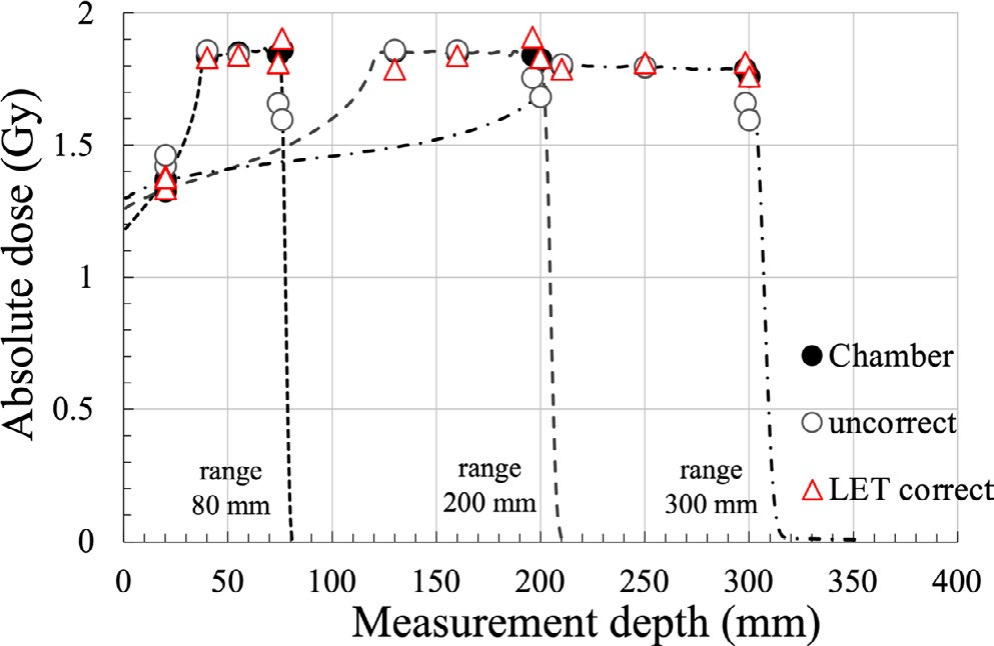
Depth dose distribution of three maximum‐range‐energy‐pattern (80, 200, and 300 mm) proton beams obtained using the IC and RGD. The dotted line represents the depth dose distribution of three maximum range energy patterns obtained by the IC during commissioning. The filled black squares, gray circles, and red triangles represent the IC, uncorrected dose, and $k_{\mathrm{LET}}^{\mathrm{RGD}}$, respectively

Figure 5a shows the dose difference as a function of R_res_. $D_{\mathrm{raw}}^{\mathrm{RGD}}$ decreased with shorter R_res_, and the under‐response for R_res_ <20 mm was 15.2% compared with the IC dose. Moreover, $D_{\mathrm{LET}}^{\mathrm{RGD}}$ was independent of R_res_, and the dose difference between the IC and $D_{\mathrm{LET}}^{\mathrm{RGD}}$ was 3.6%. Figure 5b shows the dose difference as a function of LET using the MC calculation. The dose difference between $D_{\mathrm{raw}}^{\mathrm{RGD}}$ and IC dose was within 1.3% for the LET value range of 1.4–2.8 keV/μm. The over‐response of $D_{\mathrm{raw}}^{\mathrm{RGD}}$ was 9.2% compared with the IC dose for LET values less than 1.4 keV/μm. $D_{\mathrm{raw}}^{\mathrm{RGD}}$ exhibited a linear under‐response with increasing LET, and the maximum dose difference between IC and $D_{\mathrm{raw}}^{\mathrm{RGD}}$ was 15.2% at an LET value of 6.07 keV/μm. $D_{\mathrm{LET}}^{\mathrm{RGD}}$ was independent of LET; in particular, $D_{\mathrm{LET}}^{\mathrm{RGD}}$ showed good agreement compared to the IC dose in the LET values with a range of 2–3 keV/μm. An appropriate dose could be obtained using $k_{\mathrm{LET}}^{\mathrm{RGD}}$. Figure 6 demonstrates the depth‐dose distribution of three maximum‐range‐energy‐pattern (80, 200, and 300 mm) proton beams obtained using the IC and RGD. $D_{\mathrm{LET}}^{\mathrm{RGD}}$ was consistent for all energies compared with the IC. The mean dose difference ±SD of $D_{\mathrm{raw}}^{\mathrm{RGD}}$ and $D_{\mathrm{LET}}^{\mathrm{RGD}}$ was –2.5 ± 6.9% and 0.0 ± 1.6%, respectively. The results of this study are summarized in Table 1.

**TABLE 1:** Results for the RGD dose compared to IC dose in four maximum range energy patterns

Energy, Range, SOBP width	Meas. depth (mm)	R_res_ (mm)	LET (keV/μm)	LET Uncer. (keV/μm)	Chamber dose (Gy)	Diff. uncorrect (%)	Diff. LET Co. (%)
71.6–103.1 MeV 80 mm 40 mm	20	60	0.98	0.01	1.37	6.5	0.8
	40	39	2.14	0.32	1.84	1.3	–0.2
	55	25	2.55	0.08	1.85	–0.6	–0.6
	74	5	4.98	1.44	1.84	–10.1	–1.8
	76	3	7.19	2.01	1.86	–14.3	2.3
104.5–130.5 MeV 120 mm 40 mm	20	102	0.73	0.00	1.17	9.4	2.7
	85	37	2.15	0.05	1.87	–0.1	–1.6
	100	22	2.70	0.05	1.87	0.0	0.4
	116	6	4.88	1.19	1.87	–8.2	–0.1
	118	4	6.07	1.74	1.85	–15.2	–3.4
132.3–175.7 MeV 200 mm 90 mm	20	187	0.72	0.00	1.33	7.1	0.6
	130	72	1.40	0.05	1.85	0.3	–3.6
	160	37	2.27	0.02	1.85	0.6	–0.4
	196	11	4.88	0.18	1.84	–4.7	3.6
	200	7	5.43	0.63	1.83	–7.8	0.3
178.2–221.4 MeV 300 mm 90 mm	20	291	0.79	0.00	1.37	7.0	0.7
	210	81	2.30	0.04	1.80	0.4	–0.6
	250	55	2.79	0.04	1.79	0.1	0.9
	298	13	4.94	0.30	1.79	–7.1	1.4
	300	11	5.23	0.55	1.76	–9.4	0.0

Abbreviations: Co., Correction; Diff, Difference; Meas, Measurement; Uncer, Uncertainly.

## 4 DISCUSSION

**FIGURE 7:**
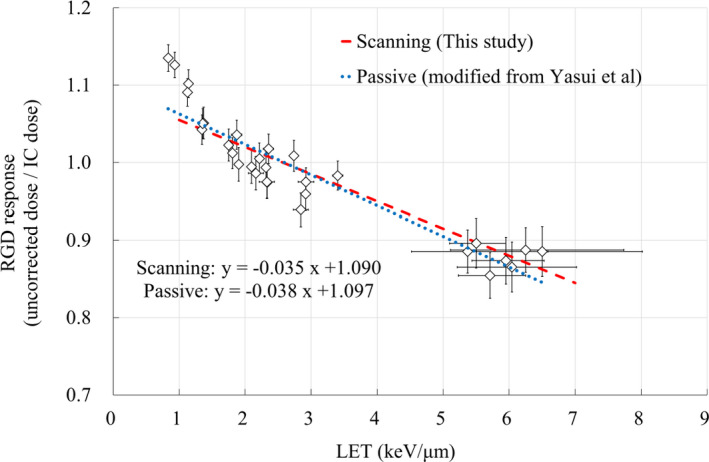
Comparison of linear approximation of RGD response as a function of LET for different irradiation methods. The red dashed line is the scanning method (this study), and the blue dotted line is the passive method (previous study). Symbols are measurement results of the passive method modified from previous study^13^

In this study, we evaluated the RGD response for a spot scanning proton beam. Because of the LET dependence of RGD, we observed a 15.2% under‐response compared with the IC dose. The LET dependence of the therapeutic proton beam was consistent with our previous study using a passive system.^13^ We obtained $k_{\mathrm{LET}}^{\mathrm{RGD}}\left(\mathrm{LET}\right)=‐0.035\left(\mathrm{LET}\right)+1.090$ from the linear fit between the RGD response and LET. The range of LET values for $k_{\mathrm{LET}}^{\mathrm{RGD}}$ was 0.72–7.2 keV/μm. The RGD response was normalized at an LET value of 2.7 keV/μm (reference condition: range 120 mm/SOBP width 40 mm), and under‐response was observed as the LET value increased. In this study, because the LET of 2.7 keV/μm is used as a reference, the LET correction factor is higher than 1.0 for LET close to 1. The LET of the calibration point, which is the basis for the correction factor, must be clarified in order to apply the $k_{\mathrm{LET}}^{\mathrm{RGD}}$ of this study to other measurements. Anderson et al. defined the response of the radiochromic film for the 70–230 MeV spot scanning system as a linear fit function of LET.^30^ A report on the LET dependence of the IC for a pencil beam scanning (PBS) carbon beam also used a linear fitting correction equation.^31^ Although these detectors show a similar trend to the present study, the LET dependence of RGD for a passive system was represented as a power function.^13^ Figure 7 shows a comparison between the results of the passive method and the results of this study. The results of the passive method are a reanalysis of the previous study.^13^ ^)^ The reference calibration point is set at LET value of 2.7 keV/μm. Applying the same linear approximation to the passive method as in scanning method, a difference between the approximate and measured values was observed in the low‐LET region, that is, at shallow measurement depths for high‐energy proton beams (160–225 MeV). We assume that this is because of the presence of many high atomic number scatterers such as range modulation wheels, second scatterers, and multi‐leaf collimators in the passive beam nozzle and the scattered radiation from these high atomic number materials had an uncertain effect at the shallow measurement depth (i.e., the low LET region). Although there is room for further research on the differences in response characteristics depending on the irradiation method, the scanning method and the passive method showed similar results, and it was suggested that the correction using the LET‐based linear approximation is effective for any irradiation method. The change in LET to R_res_ was also different between the passive and scanning systems. Therefore, each combination of the irradiation method and measurement equipment must be verified using LET.

As for a passive system, Chang et al. showed that R_res_‐based correction enables RGD‐based dosimetry with an accuracy of ±3% in the R_res_ range of 1–15 cm.^14^ In our previous study, we established that LET changes depending on the irradiation conditions, specifically for low‐energy proton beams, although R_res_ is the same.^13^ In this study, a scanning system indicated that the LET values varied by 10%–50% for the same R_res_. R_res_ is a quality index primarily established for a passive system, and it is important to use LET for a spot scanning system. However, the calculation of LET via the MC method requires long computation time and knowledge of the calculation code. Hence, there is an increasing number of reports that calculate LET using calculation or analytical methods.^32^, ^33^, ^34^, ^35^ The TPS can calculate dose and LET distributions,^36^ which can expand the LET calculation method in the future. Therefore, LET‐based correction can be realized for dose measurement using RGDs, even for advanced irradiation methods, such as intensity‐modulated proton therapy (IMPT). Furthermore, postal audits of proton facilities are usually performed under well‐defined and relatively simple conditions: LET near the center of SOBP is stable (2–3 keV/μm), and RGD is also a suitable system for postal audits if the calibration conditions are well‐defined.

The mean dose difference ±SD of $D_{\mathrm{raw}}^{\mathrm{RGD}}$ and $D_{\mathrm{LET}}^{\mathrm{RGD}}$ was –2.5 ± 6.9% and 0.0 ± 1.6%, respectively. The LET‐based correction decreased the dose difference compared to the $D_{\mathrm{raw}}^{\mathrm{RGD}}$. In this study, the uncertainty of the RGD measurement itself was not evaluated, and SD indicates the variation in the results of each measurement point. The comprehensive uncertainty of RGD dosimetry for a spot scanning system requires further investigation. The LET‐based correction can provide quantitative corrections as the quenching effect occurs because of LET.^13^ Additionally, the LET‐based correction of the RGD response is effective for a spot scanning proton beam, and the dose can be measured in distal region where accurate dosimetry is difficult. Chang et al. reported an uncertainty of 3.6% for the absorbed dose measurement of RGDs, and the uncertainty caused by LET was 3.0%.^14^ The LET‐based correction for the RGDs in our study could reduce the uncertainty caused by LET. In recent reports, in vivo measurements for a photon and a proton beam using the RGD have been reported.^37^, ^38^ We consider that LET‐based correction will lead to accurate proton dosimetry, dose audits for IMPT, and in vivo dosimetry.

The LET uncertainty was more significant in the distal region, and the maximum change of LET was 2.01 keV/μm for the 2 mm measurement depth variation assumed in this study. The details of the LET uncertainty are considered to be related to the RGD reading volume, tough water depth scaling, and effective measurement point. In this study, we calculated the LET in water and used it for correction. However, when a high‐atomic‐number material, such as the RGD, is inserted, the change in LET is yet to be determined. The change in LET with high‐atomic‐number materials will be investigated as a part of this study in the future. The feasibility of proton dosimetry using RGD is expected by further pursuing the LET‐based correction devised in this study.

## 5 CONCLUSION

In this study, we evaluated the RGD response for a spot‐scanning proton beam and devised an appropriate RGD dosimetry protocol using LET. $k_{\mathrm{LET}}^{\mathrm{RGD}}$ based on a linear function was determined from the relationship between the RGD response and LET. The LET‐based correction was consistent with the IC dose, and ±SD compared with the IC dose was 0.0 ± 1.6%. The RGD dosimetry protocol devised in this study indicated that RGD could be used for accurate proton‐absorbed dosimetry. The limitation of this study is that the LET‐based correction is highly sensitive to the measurement position, specifically in regions with steep dose gradients. Furthermore, because the RGD is constructed of high‐atomic‐number materials, the effective measurement point differs from the IC in some cases. In the future, it is necessary to evaluate the scanning proton dosimetry for IMPT and in vivo measurements using RGDs.

## CONFLICT OF INTEREST

No conflicts of interest.

## AUTHORS CONTRIBUTION

JN, KY, CO, HS, and TA were involved in study design and data interpretation.

All authors were involved in the data analysis, critically revised the report, commented on drafts of the manuscript, and approved the final report.
